# Patellar Sleeve Fracture in an Eight-Year-Old Girl

**DOI:** 10.7759/cureus.10345

**Published:** 2020-09-09

**Authors:** Mohammad O Boushnak, Mohamad K Moussa, Ahmad A Abed Ali, Zeina H Mohsen, Ali Chamseddine

**Affiliations:** 1 Orthopedic Surgery, Lebanese University, Faculty of Medical Sciences, Beirut, LBN; 2 Clinical Pathology, Lebanese University, Faculty of Medical Sciences, Beirut, LBN; 3 Orthopedic Surgery, Al-Sahel General Hospital, Beirut, LBN

**Keywords:** sleeve avulsion, patellar fracture, pediatric traumatology, pediatric sleeve fracture

## Abstract

The patellar sleeve fracture is a rare entity in pediatric traumatology. Its diagnosis is challenging due to its rarity and subtle radiographic finding, and it is easily missed by emergency physicians. Early recognition and treatment of this fracture is of paramount importance in order to guarantee better outcomes.

We present herein a case of severely displaced patellar sleeve fracture in an eight-year-old girl, which was treated successfully by open reduction and fixation of the osteochondral fragments using anchor sutures, yielding very positive clinical outcomes at the two-year follow-up.

## Introduction

Patella is a sesamoid bone, considered to be part of the quadriceps tendon and the extensor mechanism, and its ossification starts at three to six years of age [1]. The incidence of patellar fractures in children is low, at about 1% of all fractures in this population [2]. A particular type of patellar fracture occurs in skeletally immature children, called patellar sleeve fracture. This fracture involves the inferior pole of the patella, where there will be avulsion of the distal articular cartilage from the undersurface of the patella and periosteum from the upper surface, as well as complete disruption of the patellar ligament attachment [1]. The patient will present with acute knee pain, joint effusion, inability to extend the knee, and a palpable gap below the inferior pole of the patella. Plain radiographs will not usually show any fracture or bony fragment (exceptionally), except for a high-riding patella or patella alta. Thus, the diagnosis of patellar sleeve fractures is usually challenging, particularly for emergency physicians [3]. Treatment can be operative or non-operative, depending on the size of the osteochondral fragments and degree of displacement.

This paper will describe a patellar sleeve fracture that was treated operatively, with fixation of the osteochondral fragments and repair of the patellar ligament. The patient follow-up showed excellent results with no limitation of range of motion, two months after fixation and repair.

## Case presentation

An eight-year-old girl presented to the emergency room with severe right knee pain, after sustaining a fall from height on her flexed knee, landing directly on the knee in a kneeling position. Upon presentation, she had marked knee swelling with severe tenderness along the knee joint, especially when touching the patella, and a palpable gap at the inferior border of the patella. She was unable to fully extend her knee, with an extension lag around 35-40 degrees. Passive range of motion was preserved but painful throughout the arc of motion.

**Figure 1:**
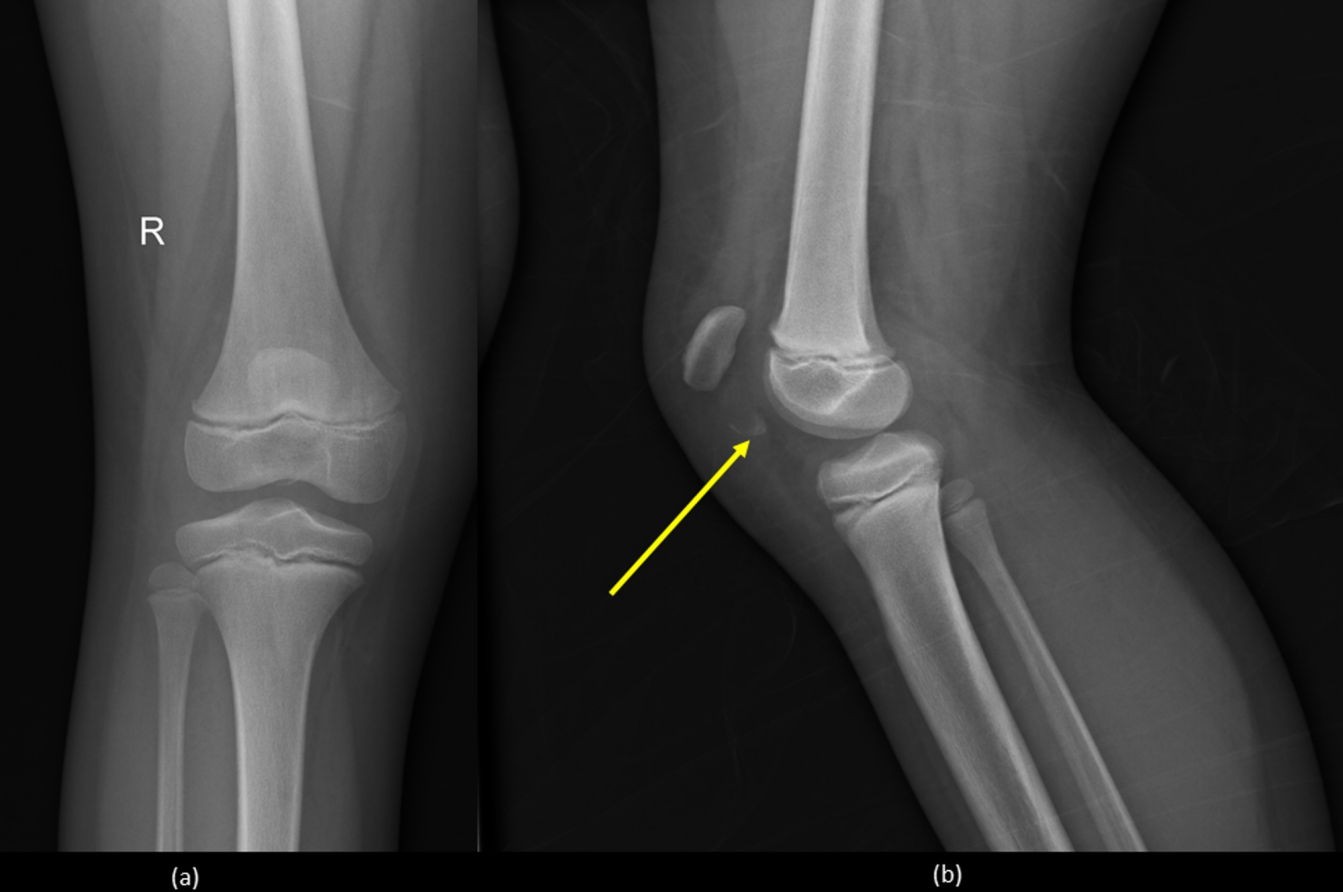
Knee radiographs. (a) Anteroposterior view and (b) lateral view showing high-riding patella with marked joint effusion, in addition to a small osteochondral fragment lying distally to the patella

Radiographs undertaken at the emergency department showed a high-riding patella with marked joint effusion, in addition to a small osteochondral fragment lying distally to the patella (Figure 1).

MRI study showed complete separation of the patellar ligament, with two large osteochondral fragments avulsed from the patella and attached to the ligament, in addition to notable hemarthrosis.

Thus, patellar sleeve fracture was diagnosed based on the high-riding patella on the radiographs, the rupture of the patellar ligament, and the avulsion of the distal osteochondral aspect of the patella. Surgical treatment was planned on the next day.

**Figure 2:**
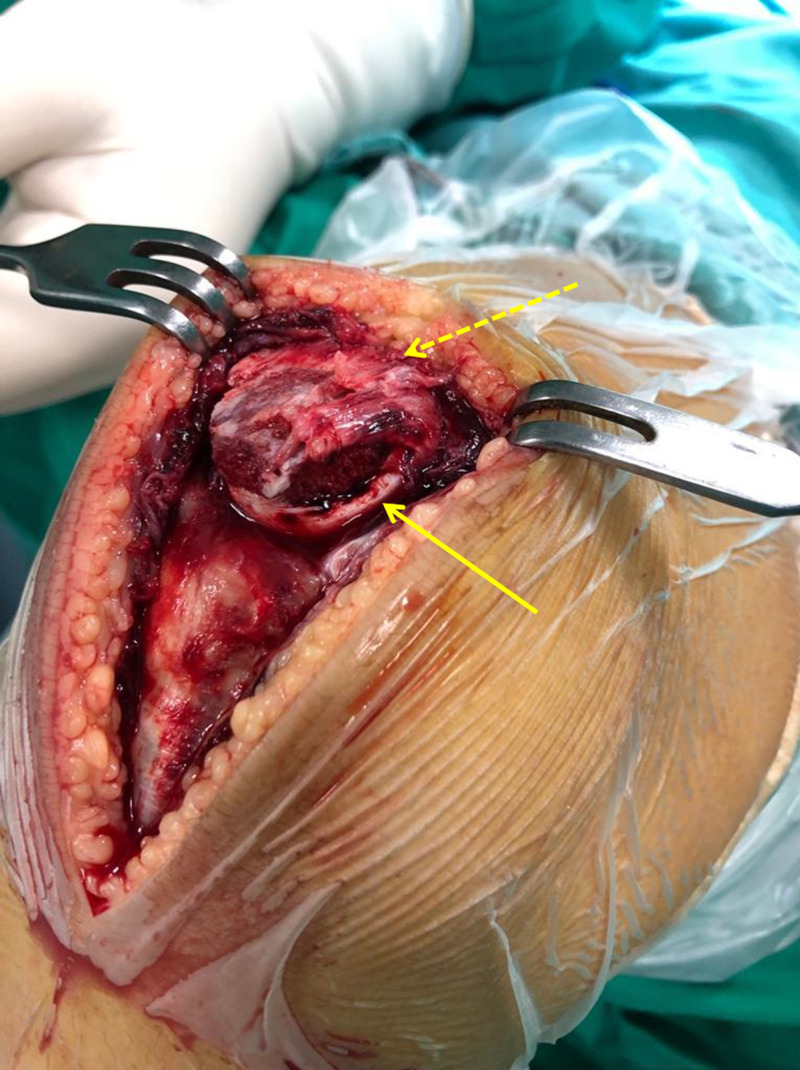
Intraoperative photograph of the knee showing complete disruption of the patellar ligament from the inferior patellar pole where about one-third of the articular cartilage (lined arrow) and half of the periosteal surface (pointed arrow) were avulsed

During the operation, complete disruption of the patellar ligament from the inferior patellar pole was noted, where about one-third of the articular cartilage (consisting of two osteochondral fragments) and half of the periosteal surface were avulsed (Figure 2).

The articular cartilage, comprising two osteochondral fragments, was large enough to get anatomically fixed with two Barouk screws. Two anchor sutures were then used to repair the patellar ligament back to the patella. Above-knee cast was then applied with 10-degree knee flexion, preventing any weight-bearing. The wound was checked regularly through a window that was created through the cast, and healing was uneventful.

**Figure 3:**
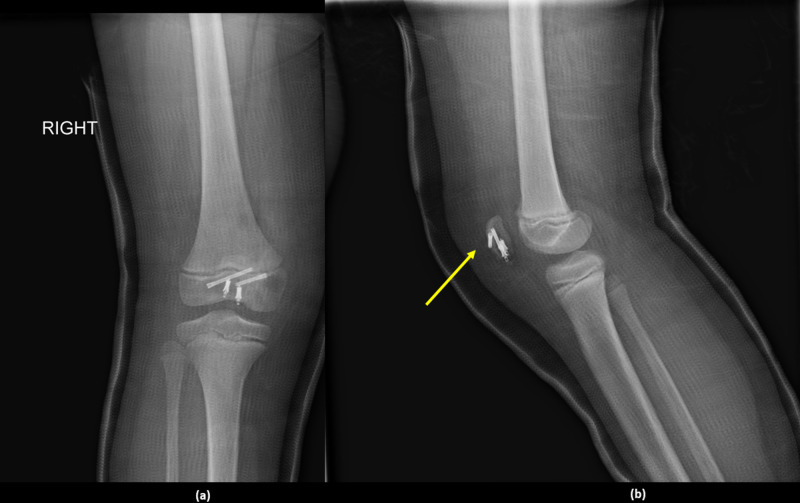
Postoperative radiographs: (a) anteroposterior view and (b) lateral view

Postoperative radiographs are shown in Figure 3.

**Figure 4:**
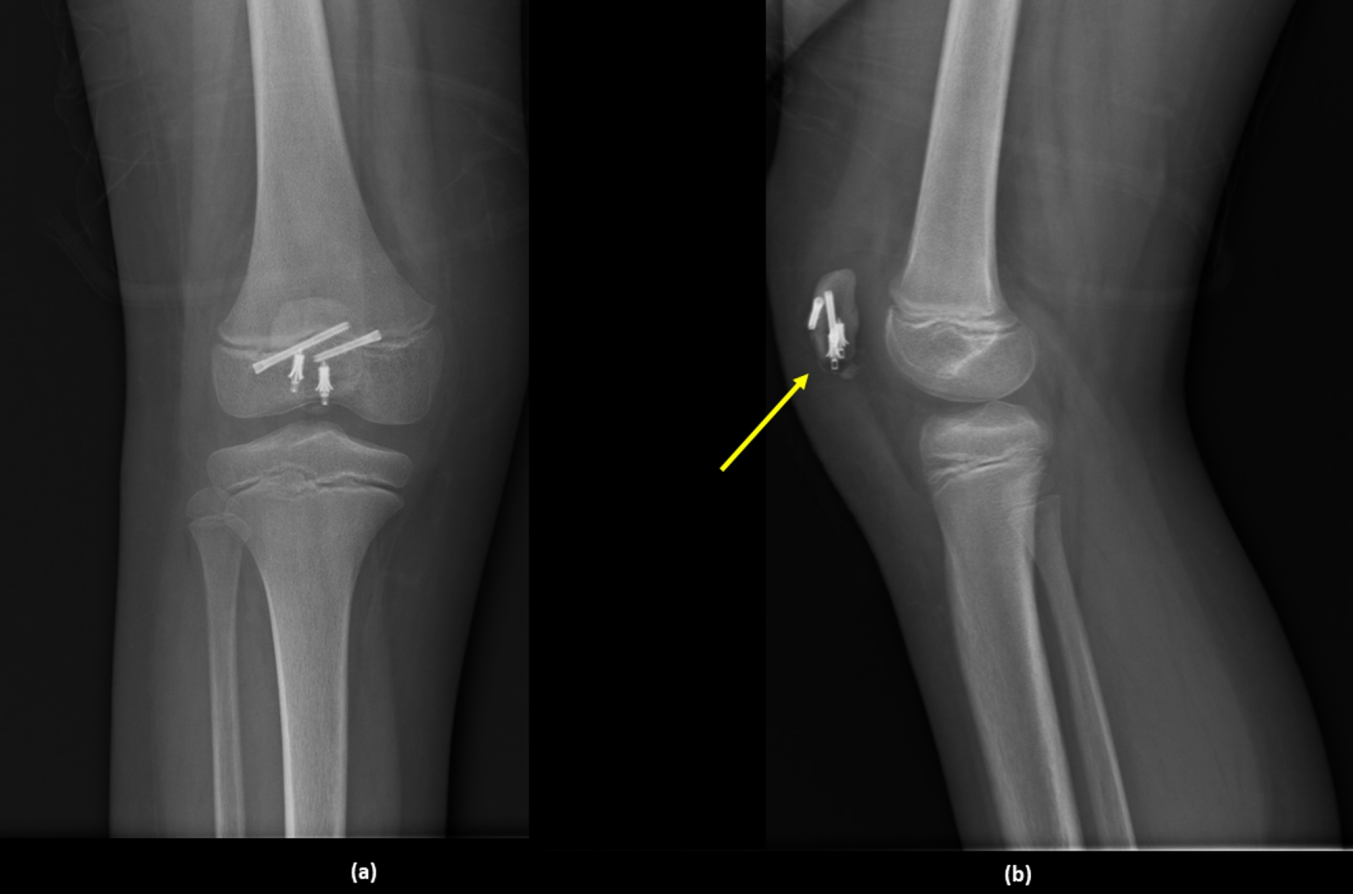
Radiographs at three-month follow-up: (a) anteroposterior view and (b) lateral view

The cast was removed after six weeks, and physical therapy started directly with full weight-bearing. One month after rehabilitation, the patient had full extension and near-full flexion of the knee joint. Radiographs at three-month follow-up are shown in Figure 4.

Two years postoperatively, the patient was symptom-free, and no long-term complications were identified.

## Discussion

The patella is a sesamoid bone of the knee that starts ossifying in a centrifugal pattern at the age of three years through multiple ossification centers (as many as six centers) [3]. Fracture of this bone is rarely documented in the pediatric age group, where the reported incidence constitutes 1% of all pediatric fractures [2].

A unique type of these patellar fractures is the patella sleeve fracture. It occurs when the fracture involves the inferior pole of the patella, causing avulsion of the distal articular cartilage from the undersurface of the patella and periosteum from the upper surface, in addition to complete disruption of the patellar ligament attachment [1]. This is the most common type of patellar fracture in children below 16 years of age, at 57% of all patellar fractures [2]. Peak incidence is at 12.7 years, with the male-to-female ratio being 3:1, respectively [4].

The mechanism of avulsion is described by rapid contraction of the quadriceps muscle on a flexed knee, resulting in an avulsion of the distal articular cartilage and periosteum from the articular and anterior surfaces of the patella, respectively [5].

Four types of patellar sleeve fracture are identified by Grogan et al: (1) superior being the least common; (2) inferior being the most common and usually associated with an acute injury as in our case; (3) medial, which can lead to lateral dislocation of the patella; and (4) lateral, which is a result of chronic repetitive stress from the vastus lateralis muscle [6].

Early identification and treatment of this condition is critical, since the avulsed fragment will lead to bone-forming tissue at the distal patella, leading to the enlargement or duplication of its size [7].

Initially, the diagnosis may be demanding and difficult, depending on both clinical and radiographic evidence deducted from physical examination, and critical analysis of the X-rays [8]. Unlike this case, where an osseous fragment was found detached from the distal pole of the patella, most patellar sleeve fractures lack this radiographic sign, and diagnosis only depends on having a high-riding patella or patella alta on the lateral plain radiograph. Patellar height is measured by the Insall-Salvati ratio, which is the length of the patellar ligament divided by patellar length on lateral X-rays. The mean ratio is 1.04 in adults (1.01 in males and 1.06 in females), with patella alta having an Insall-Salvati ratio of >1.2, and patella Baja having a ratio of <1.0 [8].

When the radiographs are not sufficiently clear to achieve a diagnosis, and patellar sleeve fracture is still a concern, other imaging modalities can be carried out to confirm the diagnosis. Ultrasound is an easy, affordable, and radiationless method that can be used; however, it is operator dependent. As in this case, magnetic resonance can also be utilized for confirming the discontinuity between the patellar ligament and the patella, as well as describing the avulsed articular cartilage that was not previously visible on plain radiographs [9].

Treatment of patellar sleeve fractures varies between conservative and non-conservative treatment, depending on the degree of displacement. Conservative treatment can be achieved with a cylindrical plaster of Paris cast immobilization, when the fracture is minimally displaced, and when the initial displacement is about 1-2 mm. Otherwise, shifting to operative treatment is a must [8].

Various techniques of open reduction are described with the aim to achieve anatomical reduction of the articular surface, including tension band wiring, trans-osseous sutures and intra-osseous anchor sutures. When the osteochondral fragment is small and rigid fixation cannot be attained, intra-osseous and trans-osseous anchor sutures can be considered [10].

Complications that might occur with patellar sleeve fracture, such as wound infection, ischemic necrosis of the patella, limitation of knee flexion due to immobilization, and muscle wasting, are the most common complications accompanying sleeve fractures [8]. Wound infections can occur, and applying an above-knee immobilizing cast postoperatively to support fixation further raises concerns. In the presented case, a window was made in the cast directly above the wound and was regularly checked for any signs of infection and healing. Ischemic necrosis of the patella can also occur, since patellar vascularization mostly arises from the anterior surface of the distal pole. Therefore, in case of any damage to the vascularization, necrosis of the proximal pole is a dreaded complication [10].

In this case, the inferior pole of the patella was divided into two osteochondral fragments that were large enough to be securely fixed with two Barouk screws. The screws were carefully inserted outside the patellofemoral joint, in addition to two anchor sutures which were also utilized for further repairing the patellar ligament to preinjury status. Above-knee cylindrical cast was applied with 10 degrees of flexion in order to achieve immobilization and protect the fixation. Although casting provides more security to the fixation, limited knee flexion due to muscle wasting after removing the cast is a known complication. Fortunately, the patient responded very well to physical therapy, where full extension and near-full flexion were accomplished rapidly, within one month after removing the cast. Although patellotibial cerclage was an option in this case, since it provides protection to the fixation, early range of motion with soon partial weight-bearing and minimal muscle wasting, it was not utilized because it might lead to patella baja and early closure of the tibial tuberosity apophysis with premature growth arrest, leading to deformity in the sagittal plane such as genu recurvatum. Add to that the pain and discomfort this cerclage can cause at the tibial apophysis [8].

## Conclusions

Ultimately, patellar sleeve fracture is a rare condition that occurs in young patients due to either a direct trauma or rapid contraction of quadriceps on a flexed knee. Identification of this condition is essential, especially in the emergency department by emergency physicians. This can be achieved through the recognition of its characteristic finding as patella alta, and hemarthrosis is of vital importance in order not to miss the diagnosis, since most of these cases present without any bony fragments that can be specified by X-rays. Non-operative treatment can be undertaken for minimally displaced fractures, whereas early surgical intervention must be carried out for displaced fractures. Fixation of large osteochondral fragments with Barouk screws and anchor sutures is an option that can be utilized safely.
